# Optimum location of external markers using feature selection algorithms for real‐time tumor tracking in external‐beam radiotherapy: a virtual phantom study

**DOI:** 10.1120/jacmp.v17i1.5861

**Published:** 2016-01-08

**Authors:** Saber Nankali, Ahmad Esmaili Torshabi, Payam Samadi Miandoab, Amin Baghizadeh

**Affiliations:** ^1^ Department of Electrical and Computer Engineering Graduate University of Advanced Technology Kerman Iran; ^2^ Department of Biotechnology Institute of Science and High Technology and Environmental Sciences, Graduate University of Advanced Technology Kerman Iran

**Keywords:** optimum location, external markers, feature selection algorithms, tumor tracking, external‐beam radiotherapy

## Abstract

In external‐beam radiotherapy, using external markers is one of the most reliable tools to predict tumor position, in clinical applications. The main challenge in this approach is tumor motion tracking with highest accuracy that depends heavily on external markers location, and this issue is the objective of this study. Four commercially available feature selection algorithms entitled 1) Correlation‐based Feature Selection, 2) Classifier, 3) Principal Components, and 4) Relief were proposed to find optimum location of external markers in combination with two “Genetic” and “Ranker” searching procedures. The performance of these algorithms has been evaluated using four‐dimensional extended cardiac‐torso anthropomorphic phantom. Six tumors in lung, three tumors in liver, and 49 points on the thorax surface were taken into account to simulate internal and external motions, respectively. The root mean square error of an adaptive neuro‐fuzzy inference system (ANFIS) as prediction model was considered as metric for quantitatively evaluating the performance of proposed feature selection algorithms. To do this, the thorax surface region was divided into nine smaller segments and predefined tumors motion was predicted by ANFIS using external motion data of given markers at each small segment, separately. Our comparative results showed that all feature selection algorithms can reasonably select specific external markers from those segments where the root mean square error of the ANFIS model is minimum. Moreover, the performance accuracy of proposed feature selection algorithms was compared, separately. For this, each tumor motion was predicted using motion data of those external markers selected by each feature selection algorithm. Duncan statistical test, followed by *F*‐test, on final results reflected that all proposed feature selection algorithms have the same performance accuracy for lung tumors. But for liver tumors, a correlation‐based feature selection algorithm, in combination with a genetic search algorithm, proved to yield best performance accuracy for selecting optimum markers.

PACS numbers: 87.55.km, 87.56.Fc

## I. INTRODUCTION

In external‐beam radiotherapy, the goal is to deliver uniform dose to tumor volume while minimizing the dose to healthy surrounding tissues. However, in thoracic region intrafractional organs motions caused by heartbeat, gastrointestinal, and especially breathing phenomena may lead to a significant uncertainty in target localization and therefore reduce treatment quality.^(1)^ in order to address this target‐localization uncertainty, several studies have been performed by suggesting improved techniques that some of them are now clinically available.^2^, ^3^, ^4^, ^5^, ^6^, ^7^, ^8^, ^9^, ^10^, ^11^, ^12^, ^13^, ^14^, ^15^, ^16^, ^17^, ^18^ An older strategy associated with this issue is to define a wider margin around gross target volume (GTV), including tumor volume and its motion trajectory, as internal target volume (ITV).^(19)^ By this approach, a great amount of high dose will be received by normal tissues inside the ITV region that may cause serious side effects. Clinically‐available strategies to compensate target motion error are: 1) breath‐holding,^8^, ^9^, ^10^, ^12^ 2) motion gated radiotherapy,^(20)^ and 3) real‐time tumor tracking.^(21)^ In the latter two cases, the patient can breathe freely during irradiation while breathing motion is monitored continuously. For this, an additional noninvasive monitoring device in combination with X‐ray imaging is needed to track tumor motion. Some of these devices include: real‐time fluoroscopy,^(2)^ electromagnetic tracking,^4^, ^16^ ultrasound,^5^, ^6^, ^18^ live MRI,^13^, ^15^, ^17^ and external surrogates.^7^, ^11^ Among these, the latter is now clinically applied due to its reliability in tumor motion estimation. There are several surrogates to correlate with tumor motion, such as: spirometer,^3^, ^22^ strain gauge,^(22)^ time‐of‐flight camera,^(14)^ ribcage,^(23)^ and external markers.^(7)^ In radiotherapy with external markers, tumor motion is correlated with external marker motion using a consistent correlation model. For this purpose, an internal fiducial maker is implanted inside or near the tumor volume, and both external and internal markers' motions are detected by infrared optical tracking and stereoscopic X‐ray imaging systems, respectively. When external–internal motion data are synchronously captured, a consistent correlation model should be configured before treatment using a synchronized training dataset. After model configuration, tumor motion may be tracked during treatment, using only external motion data as input. Several correlation models have been developed ranging from linear to non‐deterministic approaches with different performance accuracy and computational time.^24^, ^25^, ^26^, ^27^, ^28^, ^29^, ^30^, ^31^, ^32^ Comprehensive studies have been reported taking into account different aspects of available correlation models in our previous reports.^33^, ^34^, ^35^ It should be noted that the success degree of a correlation model at tumor motion prediction strongly depends on the number and location of external markers as input dataset providers.^36^, ^37^, ^38^

In most clinical practices, the location of external markers is determined empirically, which is highly operator‐dependent and is constrained by the possibility of missing optimum location. Dong et al.^(38)^ performed a mathematical study to investigate optimum markers location using Bregman distance‐based algorithm. In this study, we have proposed an alternative nonlinear strategy based on feature selection algorithms to find the best location of external markers on the thorax surface automatically, where no study has been performed before on this issue.

The feature selection concept was introduced by Liu and Motoda^(39)^ as a dimensionality reduction strategy for data mining. In this method, irrelevant features are detected and then removed to yield the most effective reduced dataset for predictive models. Accordingly, these models may avoid overtraining as a possible drawback during construction, while useless data points are removed automatically from the given dataset. Various feature selection algorithms have been proposed, with intrinsic advantages and disadvantages.^(40)^ Some of them are commercially available for various situations.^41^, ^42^, ^43^, ^44^

Four commercial feature selection algorithms were used in this work — 1) Correlation‐based feature selection (Cfs),^(45)^ 2) classifier, 3) principal components, and 4) relief^(46)^ — in combination with two Genetic and Ranker searching procedures. To do this, the Weka open‐source data mining software package dedicated on various feature selection and clustering algorithms was utilized as valuable tool.^(47)^

The required dataset used in this work was extracted from four‐dimensional extended cardiac–torso (4D XCAT) anthropomorphic phantom developed by Segars et al.^(48)^ This phantom 1) includes 3D anatomical information of different organs of human body, and 2) simulates motion of dynamic organs located in the thorax region of the patient body to mimic real respiratory and heartbeat motion patterns.

When each feature selection algorithm is used to select optimum external markers among total given markers, the motion datasets of selected markers are correlated with internal motion of a typical implanted lung or liver tumor using an external–internal correlation model. An adaptive neuro‐fuzzy inference system (ANFIS) is proposed here as a consistent correlation model to estimate tumor position information. The ANFIS model was chosen due to its proven robustness in finding reasonable complex relationship between two given datasets.^32^, ^35^ ANFIS is especially robust in situations where the dataset is not perfect and has a high degree of variability, far from presenting a regular or semi‐regular pattern.

In order to test the performance of proposed feature selection algorithms at finding optimum markers location, the thorax region was divided into several smaller segments and motion information of given external markers at each segment were utilized as input file of an ANFIS model, separately. Root mean square error (RMSE) of ANFIS output was considered as a metric tool for quantitatively evaluating the performance of proposed feature selection algorithms. In this way, the RMSE of ANFIS model output for each segment represents the “importance degree” of that segment as optimum location of external markers. Moreover, the performance of proposed strategy is compared with empirical methods that are clinically available and suggested by prior studies.^36^, ^38^, ^49^, ^50^ To do this, the RMSE of ANFIS as fed by feature selection algorithms and empirical method were compared quantitatively.

## II. MATERIALS AND METHODS

### 2.1 Dataset generation and its properties

A simulation study was performed using a commercially available NURBS‐based 4D XCAT anthropomorphic phantom to simulate motion of dynamic organs caused mainly by breathing phenomena.^(48)^ The computational XCAT phantom is based on respiratory gated 4D CT data and respiratory mechanics.^51^, ^52^ This phantom was chosen due to combined advantages of pixel‐based and geometry‐based phantoms and was quite robust to simulate human body with multiple resolutions and various anatomies. XCAT phantom enables user to change functional variables that control respiration, in order to generate deformable 4D CT models according to the real conditions that must be simulated. The main controllable parameters are: 1) motions of beating heart only, respiration only, or combined mode; 2) maximum diaphragm motion; and 3) maximum anterior–posterior expansion of the chest wall. Moreover, using this phantom, tumors with spherical shapes can be added at arbitrary organs of the simulated patient body. A deformable registration map can also be extracted by a spline‐based representation approach.^(53)^ In this study, six different respiratory cycles were generated with reasonable breathing amplitude and frequency to mimic real respiratory patterns (Table 1). For instance, maximum anterior–posterior expansion of chest wall and time of respiratory period were determined using amplitude and frequency of respiratory motion signals of real patients treated with CyberKnife Synchrony System (Accuray Inc., Sunnyvale, CA) at Georgetown University Medical Center (Washington DC).

Based on extracted dataset from XCAT phantom, since displacements of tumors and external markers in left–right (LR) direction is negligible, this dimension was eliminated from total dataset and the rest of motion dataset at anterior–posterior (AP) and superior–inferior (SI) directions were considered. It should be noted that LR motion is negligible according to 3D motion datasets of real patients treated with Synchrony CyberKnife system. During motion data extraction, the time interval between two data acquisition steps was assumed to be 25 ms.

**Figure 1:**
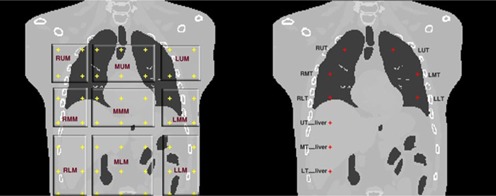
RUM=Right Upper Markers,MUM=Middle Upper Markers,LUM=Left Upper Markers,RMM=Right Middle Markers,MMM=Middle Middle Markers,LMM=Left Middle Markers,RLM=Right Lower Markers,MLM=Middle Lower Markers,LLM=Left Lower Markers. Right panel, internal tumors in lung. RUT=Right Upper Tumor,LUT=Left Upper Tumor,RMT=Right Middle Tumor,LMT=Left Middle Tumor,RLT=Right Lower Tumor,LLT=Left Lower Tumor, and in liver as: UT_liver=Upper Tumor in liver,MT_liver=Middle Tumor in liver, LT_liver=Lower Tumor in liver.

In order to assess optimum external markers location, several points were defined 1) on the thorax region of phantom surface as external markers, and 2) inside lung and liver organs as internal markers representing tumor location. Forty‐nine points were uniformly distributed onto the surface of the chest and abdominal regions of the phantom, each of them representing external surrogates. The scheme of depicted points was started from the abdominal region, averaging 5 cm distance in vertical and horizontal directions (Fig. 1, left side). As seen in this figure, the proposed spatial scheme of given points was divided into nine smaller regions including four to nine external markers.

Concerning tumors simulation, three points in the right lung and three points in the left lung were considered in upper, middle, and lower lobes of lung (Fig. 1, right side). Moreover, three points were assumed to be the origin of tumors located at lower, middle and upper lobes of liver.

**Table 1:** Characteristics of six different respiratory cycles created by XCAT Phantom.

*Maximum Anterior–Posterior Expansion of Chest Wall (CM)*	*Maximum Diaphragm Motion (CM)*	*Time of Respiratory Period (S)*	*Breathing Cycle Number*
1.2	2	5	1
0.7	1.7	5	2
0.5	1.2	4	3
1.3	2.2	6	4
1	1.8	5.5	5
0.5	1	3.5	6

### 2.2 Feature selection algorithms

Feature selection is introduced as useful available technique required at data preprocessing step for data mining. In this technique the number of features (the external markers, in this work) is reduced by removing irrelevant, redundant, or noisy data. Therefore, most effective and remarkable data extracted through total dataset in this strategy can improve the performance accuracy and result in a better assessment condition.

In order to find best location of external markers, four feature selection algorithms were employed using the Weka software package.^(47)^ Table 2 lists these algorithms including feature evaluation and searching methods. In this Table, the last two rows process total features with corresponding searching algorithm on a case‐by‐case basis and remove the unnecessary features. For this purpose, a test criterion was predefined to remove unnecessary features. The other feature selection methods illustrated in this table evaluate the space of feature subset searched by the proposed Genetic searching method. The last two methods shown in Table 2 are potentially faster, with less performance accuracy.^(54)^

The proposed feature selection algorithms are briefly described below:

1. “Correlation based Feature Selection Subset Evaluation” considers predictive value of each feature individually along with the degree of redundancy among them.^(45)^
2. “Classifier Subset Evaluation” uses a classifier to evaluate sets of features in the training data. In our study, the classifier used for estimating the accuracy of subsets was “zeroR,” which predicts the average value for a numeric class.
3. “Principal Components” were used for analysis and transformation.
4. “ReliefF Attribute Evaluation” evaluates the worth of a feature by repeatedly sampling an instance and considering the value of the given feature for the nearest instance of the same and a different class.^(55)^

Apart from the four proposed feature selection algorithms shown at Table 2, there are two searching procedures known as Genetic and Ranker searches. The proposed Genetic algorithm uses a simple genetic algorithm^(56)^ and the Ranker search method ranks features by their individual evaluations. Genetic algorithms (GAs) use a heuristic searching algorithm that works according to concepts of natural selection and genetics. In this method, useful solutions for data optimization and searching processes emerge.^(56)^

Moreover, before using feature selection algorithms, in order to reduce the number of inputs, principal components analysis (PCA) was implemented for two Y and Z variables, and then the first principal component was utilized. Principal component analysis (PCA) statistically transforms a set of potentially correlated variables into linearly uncorrelated variables, called principal components, that may be equal to or less than the number of original variables. After transformation, the first principal component has the largest possible variance among all generated principal components.^(57)^ By applying PCA algorithm, a small amount of data will be lost when the first component of all markers covers more than 90% of variance. PCA transforms the 2D motion data of external markers into a monodimensional signal, by projecting the two‐dimensional coordinates in the principal component space.

**Table 2:** Feature evaluation methods associated with proposed searching methods.

*Feature Selection Algorithm Number*	*Feature Evaluation Method for Feature Selection*	*Search Method for Feature Selection*
1	CfsSubsetEval	Genetic Search
2	ClassifierSubsetEval	Genetic Search
3	Principal Components	Ranker
4	ReliefFAttributeEval	Ranker

CfsSubsetEval=Correlation based Feature Selection Subset Evaluation;ClassifierSubsetEval=Classifier Subset Evaluation;ReliefFAttributeEval=Relief Attrifute Evaluation

### 2.3 Data analysis and feature selection criteria using ANFIS correlation model

At first, the motion data of external–internal markers was fed to PCA algorithm to reshape AP and SI motion data format from 2D Y‐Z information into a monodimensional signal containing both AP and SI motion information. It should be noted that the performance of feature selection algorithms (without PCA implementation) is based on most effective motions data on Y and Z independently, without considering to find a compromise between both Y and Z motions data for a typical external marker. This issue is important for us because we are looking for an optimum external marker, not its motion properties only on Y or Z direction.

Hence, PCA analysis was used in order to select the best markers with whole‐motion information.

The external motion data processed by PCA was then given to proposed feature selection algorithms. Therefore, the output of PCA signal, which is monodimensional, is the only input dataset for all feature selection algorithms. Taking into account nine tumors, a total of 9×4=36 computational processes were calculated. Required motion data for feature selection algorithms was taken from breathing cycle data shown at Table 1.

Moreover, in order to investigate the performance of proposed feature selection algorithms to discover optimum markers location, an alternative method was used based on an external–internal correlation model as benchmark. In this strategy, at first, without considering feature selection algorithms, motion information of external markers was correlated with internal motion data and the best correlation represents the best placement of external markers. Then we can compare best placement of external markers with markers selected by feature selection algorithms. For this purpose, the thorax surface was divided into nine smaller segments, as shown in Fig. 1 (left side). In this figure, each segment includes four to nine points representing external markers. For nine small segments on the thorax surface external markers of each segment must be correlated with nine lung and liver tumors using ANFIS model. Therefore, total number of models running is 9×9=81. It should be noted that the input data of ANFIS were already processed by PCA algorithm to reduce data dimensionality and to avoid computational complexity and model overtraining. For a given tumor, the best segment is chosen according to the least RMSE of ANFIS model output among nine calculations. The procedure was used for nine tumors with same calculations and the best segment was selected for each tumors. It should be noted that for each calculation, ANFIS model was executed ten times and the average of RMSE was reported as the final value. RMSE between benchmarked and model output was calculated according to the following metric:

(1) $$RMSE=\sqrt{\frac{1}{N}\sum_{i=1}^{N}{\left(A_{i}-P_{i}\right)}^{2}}$$

where, *N* is the number of predicted samples, $A_{i}$ is *i*th output in the dataset as real position information, and $P_{i}$ is the *i*th predicted output by the model.

As the next step, the results of ANFIS model were compared with results of four feature selection algorithms in finding optimum markers placement. Furthermore, in order to select the best performance among feature selection algorithms, 2D movement dataset of the chosen markers by each algorithm was given to ANFIS model and then model outputs were compared with each other, quantitatively.

**Figure 2:**
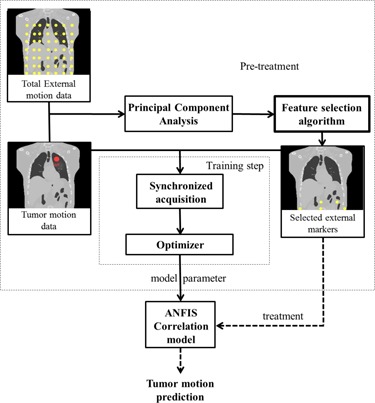
Flowchart of a typical tumor motion prediction by ANFIS using optimum external markers chosen by a typical feature selection method in combination with PCA preprocessing algorithm.

Figure 2 shows a schematic layout of 1) Principal Component Analysis in data processing requirement of feature selection algorithm, and 2) ANFIS correlation model configuration at pretreatment step and model performance during treatment. When optimum external makers were selected, their motion data were as synchronized with internal tumor motion for model constructing. As depicted at the dashed smaller rectangular in this figure, ANFIS configuration is done at training step using synchronized external–internal training dataset for determining model parameters. After configuration, the model can infer tumor trajectory using only motion data of the selected external marker.

Moreover, in order to compare our strategy with currently used empirical methods, tumor motion was predicted by means of five external markers located on abdominal, diaphragm, and above‐tumor sites as common available locations for external markers in clinical applications.^36^, ^38^, ^49^, ^50^

In summary, to find the optimum spatial pattern of external markers, an alternative nonlinear strategy based on feature selection algorithms was used. Four commercially available feature selection algorithms were utilized as comparative study using two Genetic and Ranker search methods. After implementing feature selection algorithms, the output of each method was considered as required external motion dataset for ANFIS correlation model. The RMSE of ANFIS output was taken into account as test criterion for evaluating our proposed strategy. The required motion dataset was provided by means of 4D XCAT anthropomorphic phantom.

### 2.4 ANFIS correlation model

Generally, when the system is working properly there is a reasonable relationship between several datasets generated by this system as output. This relation may be simple (linear) or complicated (nonlinear) depending on system complexity. For nonlinear systems, several models are proposed to correlate two datasets with different parameters extracted from a unique system. These models work on the basis of nondeterministic mathematical rules to estimate proper correlations between datasets with an uncertainty error.

In this work, we utilized our ANFIS correlation model by implementing the fuzzy logic toolbox of MATLAB (MathWorks Inc., Natick, MA).^(58)^ ANFIS is presented as a powerful tool for modeling numerous processes by combining the abilities of a fuzzy system with the numeric power of neural network system. It extracts fuzzy rules from numerical data with a highly variable range of input/output dataset and adaptively constructs a rule base, where calculations are impossible or difficult using normal mathematics approaches. In this study, we used the robustness of ANFIS particularly as a correlation model for respiratory motion tracking where the same calculations with normal mathematical methods would be difficult or less accurate, due to the high degree of uncertainty of breathing phenomena. Our ANFIS correlation model must be trained using external–internal motion data at synchronized form in training step. After configuration, the model is able to track tumor motion as a function of time using only external motion data. Model training was done by motion information of 5 phases and the last phase was used for model test (Table 1).

## III. RESULTS

In this work, five external markers were assumed to make proper correlation with internal tumor motion. It should be considered that the number of utilized external markers is clinically flexible ranging from three to five. According to Fig. 1, each external marker belongs to a specified segment as depicted on thorax region.

Generally, each segment has its own degree of importance due to two major factors: 1) correlation of a corresponding marker with tumor motion, and 2) its motion amplitude during respiration. In the proposed algorithm, this degree of importance is increased when a large number of external markers belonging to a typical segment are selected by our proposed feature selection algorithms.

The number of optimum external markers from a given segment was selected by each feature selection algorithm. The selection procedure was repeated using all proposed methods and then the summation of all chosen external markers at each segment was calculated for each tumor, individually (Table 3). This summation value can represent the importance degree of each segment. For example, to predict LLT, the number of selected markers from LLM segment was two by CfsSubsetEval, one by ClassifierSubsetEval, one by Principal Components, and none by ReliefFAttributeEval. Therefore, the summation of all selected markers from this example is four.

According to Table 3, without considering tumor location, the most important segments are middle lower and right lower for optimum marker selection as predicted by all feature selection algorithms. In other words, three lower segments have the highest degree of importance where six upper and middle segments with lower values are far away from participation as optimum external markers.

**Figure 3:**
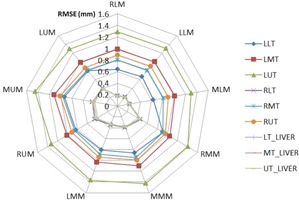
Root mean square error of tumor motion prediction via ANFIS model, using external markers from each segment, for all tumors.

In order to validate feature selection algorithms, the motion of each tumor was separately predicted by feeding ANFIS model using specific markers of each segment directly without using feature selection algorithms. The same calculations were done for all tumors placed at nine segments using ANFIS model for tumor motion prediction. The results are shown in Fig. 3 as a radar plot that illustrates RMSE calculated between ANFIS model output and the real position of each tumor. As seen in this figure, lower segments including LLM, MLM and RLM have the least RMSE in comparison with other segments for each tumor. Therefore, the mentioned segments are determined as proper location for external markers in order to make consistent correlation with tumor motion. Taking into account results of Table 2, segments with highest importance degree proposed with our algorithm are exactly same as segments with least RMSE indicated in Fig. 3. This means that the performance of proposed feature selection algorithms is reasonable at finding proper segments to select external markers.

**Table 3:** Total number of external markers for each segment onto thorax surface selected by proposed feature selection algorithms.

*UTLiver*	*MTLiver*	*LTLiver*	*RUT*	*LUT*	*RMT*	*LMT*	*RLT*	*LLT*	*Tumor Marker*
3	3	3	3	4	4	4	3	4	LLM
7	7	7	6	7	6	6	8	7	MLM
7	7	7	8	6	7	7	6	6	RLM
0	0	0	0	0	0	0	0	0	LMM
2	2	2	2	2	2	2	2	2	MMM
0	0	0	0	0	0	0	0	0	RMM
1	1	1	1	1	1	1	1	1	LUM
0	0	0	0	0	0	0	0	0	MUM
0	0	0	0	0	0	0	0	0	RUM

**Figure 4:**
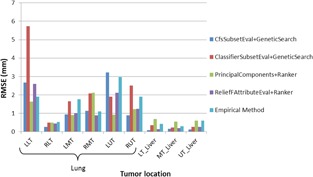
Root mean square error of tumor motion prediction via ANFIS using all feature selection algorithms from Table 2 and without using any feature selection algorithm (Empirical Method), for all tumors.

As seen in Fig. 3, liver tumors can be predicted with less error compared to lung tumors. In addition, among lung tumors maximum and minimum prediction errors belong to tumors located at upper lobe of left lung (LUT) and lower lobe of right lung (RLT), respectively. Figure 4 shows prediction errors of predefined tumors, using ANFIS model fed by markers located empirically and selected automatically by feature selection algorithms.

**Figure 5:**
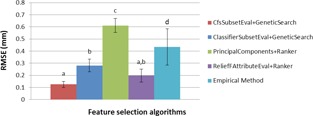
Duncan test to compare mean error of liver tumors motion prediction using four feature selection algorithms, and without using them (Empirical Method).

In order to perform non‐parametrical statistical analysis, an F‐test was applied to compare the performances of feature selection algorithms and empirical method. Furthermore, a Duncan test was implemented to evaluate the mean error of these algorithms. The results of two statistical tests were shown at Fig. 5.

Based on Duncan test for liver tumors (Fig. 5), except for the Principal Components feature evaluation method in combination with Ranker search method, which has the worst performance among the four proposed algorithms, other feature selection algorithms had better performance than the empirical method. Furthermore, the best performance results by using the correlation‐based feature selection subset evaluation in combination with the Genetic search method. Quantitatively, the ratio of RMSE by CfsSubsetEval method to Principal Components, ClassifierSubsetEval, ReliefFAttributeEval, and empirical method are 0.2, 0.4, 0.6, and 0.3, respectively. For lung tumors, based on Duncan statistical test, the accuracy of all feature selection algorithms is almost same in an acceptable range and better than empirical method.

## IV. DISCUSSION

In radiation treatment, many tumors located in thorax and abdomen regions move semi‐regularly, mainly due to respiration. This motion is problematic during therapeutic beam irradiation, and may result in undesirable dose distribution on tumor volume and may also deliver high dose to healthy non‐target tissues. Several efforts have been made to compensate this intra‐fractional motion error. Among them, continuous X‐ray imaging may be ideal for tumor motion monitoring but a large amount of additional imaging dose received by patient is a serious concern according to ALARA principles. As a solution, external surrogates‐based radiotherapy has been proposed to minimize additional imaging dose. In this approach, the internal tumor motion is correlated with the external thorax surface using a proper internal–external correlation model.

In this strategy, the success of the correlation model in tumor motion tracking is strongly affected by the number and location of external markers. In most clinical practice, the location of external markers is chosen empirically; that is, operator‐dependent. Moreover, few studies have been performed to mathematically investigate optimum location of external markers.

In this study we presented a general framework that comprehensively investigates the effect of feature selection algorithms in external markers placement on the performance of correlation model using XCAT as an available anthropomorphic phantom. Using the XCAT phantom, internal organs at thorax region such as lung and liver are easily accessible to define any arbitrary tumors at different sites with different sizes where there is no option in real conditions for undertaking the same investigation. In contrast, since simulating different organs with detailed information and motion issues are not exactly the same as in a real patient body due to phantom simplification, some uncertainty errors may arise during phantom performance. But these mismatching errors are reasonable within an acceptable range. In this phantom, the parameters of respiratory cycles and diaphragm motion can easily be changed as well to simulate various displacements that posed a limitation in the prior study.^(38)^

Nine tumors (three in liver and six in lung) with 49 points representing external markers were defined using XCAT phantom to generate comprehensive datasets for finding optimum external markers location. Moreover, six different respiratory cycles with different patterns were constructed in order to simulate different respiratory conditions close to motion data of real patients. Four feature selection algorithms in combination with two searching methods were proposed in this work to automatically select optimum location of external markers using the Weka software package.

Final analyzed results represented that all proposed feature selection algorithms work reasonably at selecting proper location of external markers. As shown in Table 3 and Fig. 3, there is a good agreement between the number of selected external markers of each segment and corresponding RMSE of ANFIS from that segment. But, in detail, for liver tumors, the Correlation‐Based Feature Selection Subset Evaluation method in combination with the Genetic search method has the best performance.

As illustrated in Fig. 4, for each tumor motion prediction there is at least one feature selection algorithm that has better performance (less RMSE) than the empirical method. Therefore, feature selection algorithms are proved to increase treatment quality by selecting optimum locations of external markers.

Comparing lung tumors vs. liver tumors it is worth mentioning that total lung tumors have larger prediction error using ANFIS against total liver tumors by a ratio of 2.7:1. Among lung tumors, LUT and RLT have maximum and minimum prediction error, respectively.

The ratio of ANFIS output error using external markers located at lower segments versus the two middle and upper segments was around 0.8. However, 85% of markers selected by the four feature selection algorithms belonged to the lower segment, while this percent was 10% and 5% for middle and upper segments, respectively. Moreover, the ratio of motion amplitude for lower segment to middle and upper segments was 1.06 and 1.3, respectively. Therefore, the great numbers of external markers were selected from the segments which had largest motion amplitude and highest correlation with tumor motion. It should be noted that using these two parameters (amplitude and correlation) is a typical way of marker placement for tumor motion tracking in clinical practice.^(38)^ This fact showed the robustness of feature selection algorithms in detecting optimum location of external markers, as is the main purpose of this study.

It should be considered that the relation between selected external markers motion and tumor motion was independent of the distance between markers locations and tumor site.

Future studies can be performed in investigating feature selection algorithms adaptively to determine number and location of external markers in real patient data using deformable image registration techniques.^(59)^ In this work thoracic 4D CT or CBCT data with several phases will be used. A mesh of pixels (points) will be determined on the patient's thorax in a reference phase and propagated to other phases using deformable image registration of the relevant CT (CBCT) images. Moreover, an intelligent model will be used to find the best feature selection algorithm between numbers of them.

## V. CONCLUSIONS

In this study, we used a 4D XCAT phantom to investigate several feature selection algorithms to find optimum external marker locations which have the best correlation with internal tumor motion. We realized that feature selection algorithms have better performance in regard with empirical method. The best feature selection algorithm for liver tumors was the CfsSubsetEvalfeature evaluation algorithm in combination with the Genetic search algorithm, which offers best marker locations to predict tumor motion using ANFIS model. For lung tumors, all four proposed feature selection algorithms had reasonable performance with similar precision. Most of the markers are selected from the segments with largest motion amplitude that have highest correlation with tumor motions. Finally, though the study here focuses on optimal marker placement using XCAT phantom, the proposed concept can be implemented on real patient data in our future studies.

## ACKNOWLEDGMENTS

The authors acknowledge Dr. William Paul Segars and Sonja Dieterich for providing access to the Anthropomorphic XCAT phantom and clinical Cyberknife database, respectively.
